# Can Virtual Reality Cognitive Rehabilitation Improve Executive Functioning and Coping Strategies in Traumatic Brain Injury? A Pilot Study

**DOI:** 10.3390/brainsci13040578

**Published:** 2023-03-29

**Authors:** Rosaria De Luca, Mirjam Bonanno, Angela Marra, Carmela Rifici, Patrizia Pollicino, Angelo Caminiti, Milva Veronica Castorina, Andrea Santamato, Angelo Quartarone, Rocco Salvatore Calabrò

**Affiliations:** 1 IRCCS Centro Neurolesi “Bonino Pulejo”, 98123 Messina, Italy; rosaria.deluca@irccsme.it (R.D.L.); angela.marra@irccsme.it (A.M.); carmela.rifici@irccsme.it (C.R.); patrizia.pollicino@irccsme.it (P.P.); angelo.caminiti@irccsme.it (A.C.); milva.castorina@irccsme.it (M.V.C.); angelo.quartarone@irccsme.it (A.Q.); roccos.calabro@irccsme.it (R.S.C.); 2 Department of Clinical and Experimental Medicine, University of Foggia, 71122 Foggia, Italy; a.santamato@unifg.it

**Keywords:** TBI, executive dysfunctions, coping strategies, virtual reality, cognitive rehabilitation

## Abstract

Executive dysfunction is among the most common and disabling facets of cognitive impairment following traumatic brain injury (TBI), and may include deficits in reasoning, planning, mental flexibility, some aspects of attention and orientation, awareness and behavior. Rehabilitation programs based on cognitive-behavioral approaches to retrain planning and problem-solving and other executive deficits may improve such cognitive dysfunction. The purpose of this study is to investigate the effects of non-immersive virtual reality-based training to improve executive abilities and to reduce anxiety and depression symptoms in patients with TBI. Twenty patients with moderate to severe TBI were enrolled at our Neurorehabilitation Unit and divided to receive either the standard cognitive training or the virtual reality (VR) based cognitive training using the virtual reality rehabilitation system (VRRS-Evo). Each group received the same amount of rehabilitative training, including ROT (Reality Orientation Therapy) and Executive Training (ET), but using a different approach, i.e., a paper and pencil and an advanced approach. All patients were evaluated with a specific psychometric battery before (T0) and after the end (T1) of each program. Comparing pre- and post- treatment scores, in the VR-CT group, we found statistically significant differences in all administered outcome measures for cognitive and executive functioning, i.e., MoCA (*p* < 0.005), FAB (*p* < 0.005), TMT-A (*p* < 0.005), TMT-B (*p* < 0.005), TMT-BA (*p* < 0.001), and mood, i.e., HRS-D (*p* < 0.008). In the Conventional cognitive training (C-CT) group, we found a significant improvement only in MoCA (*p* < 0.03), FAB (*p* < 0.02) and in TMT-BA (*p* < 0.01). Coping strategies also improved, with better results in the VR-CT group. Our results suggest that VR rehabilitation, using the VRRS system, may be a valuable and motivational approach to improve visuo-executive abilities and coping strategies as well as mood in chronic TBI patients.

## 1. Introduction

Traumatic brain injury (TBI) affects more than 200 per 100,000 people each year globally [1,2], and it may cause permanent or temporary cognitive dysfunctions, motor and behavior deficits as well as chronic disorders of consciousness. Post-TBI disability is related to the site and the extent of the brain damage [3] and patients must deal with their daily concerns for a very long time. Generally, individuals after a TBI use two main sets of coping strategies, either adaptive or maladaptive, and this depends on their personality trait and cognitive status [4]. In fact, a strong association between deficit in executive function, especially problem solving, and coping styles has been reported [5,6]. Executive dysfunction (ED) is an “umbrella term” which includes several cognitive processes, such as decision-making, impulse control, attention, behavioral flexibility, organization and working memory [7,8,9], which are often associated with neurobehavioral abnormalities, including depression symptoms and mood alterations [10]. Commonly, ED can appear both in the acute and the chronic phase, involving about 57% of TBI patients [11]. It has been shown that damage in the orbito-frontal and ventro-medial brain areas could particularly involve the so-called “hot” executive functions, which include reward, emotion and motivation; brain injuries to the dorso-lateral regions are indeed associated with alterations of the “cold” executive functions, which involve cognitive information processing, such as working memory [12]. Cognitive rehabilitation (CR) is a systematic, functionally oriented therapeutic intervention, based on a patient’s cognitive and behavioral alterations [13]. Specifically, the Reality Orientation Program (ROP) is a CR technique used to improve orientation, social skills and awareness, as well as the executive functions. The training sessions can be performed individually (the “informal” approach) [14] or in groups (the “formal” approach) [15], involving stimulation and repetition of basic information [16]. The use of ROT is commonly administered in patients with TBI, even in the early stages of their recovery [17,18]. Limited evidence has so far shown that standard CR and specific rehabilitative methods are effective in the treatment of cognitive alterations in chronic TBI [19,20]. The last decade has witnessed a growing interest in what technology can do to better manage TBI patients. In detail, innovative tools have been implemented in clinical practice to stimulate cognitive recovery following TBI [21,22]. However, a few studies have shown the effectiveness of cognitive training using virtual reality (VR) [21,22,23]. VR tools offer the possibility to adapt the exercises to the patient’s capabilities and needs and monitor their performance. VR may increase patients’ motivation and active participation thanks to the visual and auditory feedback. Then, VR can be properly used to train different cognitive domains, including executive functions, in TBI patients. 

The purpose of this pilot study is to evaluate the effects of a non-immersive VR-based cognitive training focusing on potentiating cognitive recovery and improve coping strategies and mood in chronic TBI subjects. 

## 2. Material and Methods

### 2.1. Study Population 

Twenty patients affected by moderate to severe chronic TBI (i.e., at least 6 months after the event) with a mean age of 44.6 ± 16.13, having attended from October 2021 to March 2022 the Outpatient clinic of the Neurorehabilitation Unit of the IRCCS Neurolesi “Bonino-Pulejo” of Messina, were enrolled in this study. Either TBI patients and/or their caregivers were adequately informed about the study and offered their collaboration and written consent. The study was performed following the Helsinki declaration of human rights, and the local Ethics Committee approved the study (IRCCS-ME-CE 08/21).

A more detailed description of the two groups is listed in Table 1.

### 2.2. Procedures

TBI patients were randomly assigned to one of two groups using a web-based application for block randomization (www.randomization.com, accessed on 12 January 2022). We used the block randomization method (block size = 4) to ensure balance in the sample size across groups over time. The experimental group received the innovative training for executive function (VR-CT; 5 male patients and 5 female patients with a mean age of 46.2 ± 14.9 years), and the control group (C-CT; 6 male patients and 4 female patients, mean age 43.1 ± 17.9 years) was submitted to a conventional cognitive treatment.

Patients were enrolled according to the following inclusion criteria: (i) diagnosis of moderate to severe TBI in the chronic phase (≥6 months from the traumatic event); (ii) presence of moderate to severe cognitive alterations due to TBI (i.e., MoCA ≥ 17; (iii) while exclusion criteria were: epilepsy, disabling sensory alterations (including visual and hearing deficits) and medical illness, severe cognitive and behavioral disturbances which could interfere with the cognitive training.

A blind to patient’s allocation neuropsychologist administered a psychometric battery, before (T0) and after (T1) the executive function training. The cognitive and psychometric evaluation included global cognitive function as well as specific neuropsychological tests to evaluate cognition and executive abilities. 

All study participants, after the neuropsychological evaluation, received the same standard cognitive rehabilitation, 3 times a week for 8 weeks (i.e., 24 sessions of 60 min each). In addition, the VR-CT group was trained with the virtual reality rehabilitation system (VRRS) (24 sessions of 60 min each, 3 times a week for 8 weeks) to potentiate executive function, while the controls performed the same amount of standard CR for the executive deficits (24 sessions, 3 times a week for 8 weeks) (Table 2). 

### 2.3. Outcome Measures 

The psychometric battery included: (i) Montreal Cognitive Assessment (MoCA) [24], a rapid cognitive screening to assess specific sub items such as attention processes, executive functioning, memory functions, language, visuo-constructional abilities, thinking, calculations, and orientation; (ii) Trail Making Test (TMT) that measures attention process, visual search and scanning, sequencing and shifting, psychomotor speed, abstraction, and flexibility and executive functions [25]; (iii) Frontal Assessment Battery (FAB) that consists in a short neuropsychological tool aiming to assess executive functions, including S-word generation, similarities, Luria’s test, grasp reflex, and the Go-No-Go test [26]; and (iv) Hamilton Rating Scale for Depression (HRS-D) investigating the presence and level of depression symptoms [27] to avoid confounding factors in cognitive training and recovery. In addition, coping strategies were evaluated through the Coping Orientation to the Problems Experiences-new Italian version (COPE-NIV), which consists in a self-report questionnaire, used to quantify effective and ineffective ways to cope with a stressful life event. The COPE-NIV included five large essentially independent dimensions: social support, avoidance strategies, positive attitude, problem solving and turning to religion [28]. 

### 2.4. Conventional Cognitive Training (C-CT)

The standard treatment focused on executive processes and was based on a face-to-face approach between the therapist and the patient using paper and pencil tools and other traditional materials. It was mainly aimed at strengthening orientation with a specific cognitive program using the reality orientation therapy (ROT), including tasks for specific cognitive domains: autobiographical memory, temporal and spatial/topographic orientation, and simple relationships and logic associations were also trained. The training of the visuo-executive abilities was carried out by working on categorization, planning, association processes, analogical reasoning, problem solving and coping strategies to simulate problematic situations in a protected context, thanks to a constant interaction with the cognitive therapist (see Table 3). 

### 2.5. VR Cognitive Training (VR-CT)

The VR training also focused on potentiating executive processes. It was developed to stimulate the same cognitive domains as the C-CT, but using the innovative tool (Table 3).

**Figure 1:**
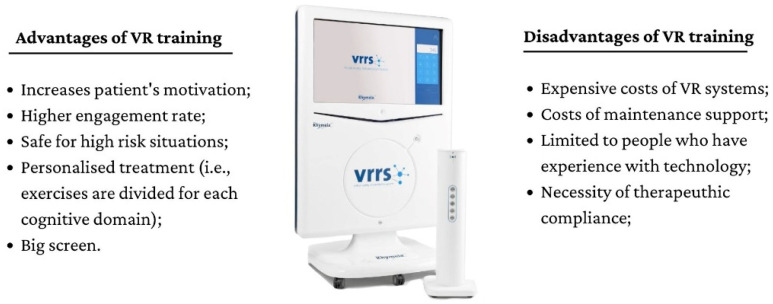
The virtual reality system used to train traumatic brain injury patients showing both advantages and disadvantages about its use in cognitive rehabilitation.

The VRRS (Figure 1) is a valuable device providing various virtual exercises divided into different modules, such as: motor (postural, upper and lower limbs activity, facial expressions and respiratory exercises), logopedic and cognitive, whereas exercises are divided into specific domains (i.e., executive functions, attention, praxis and so on…). 

**Figure 2:**
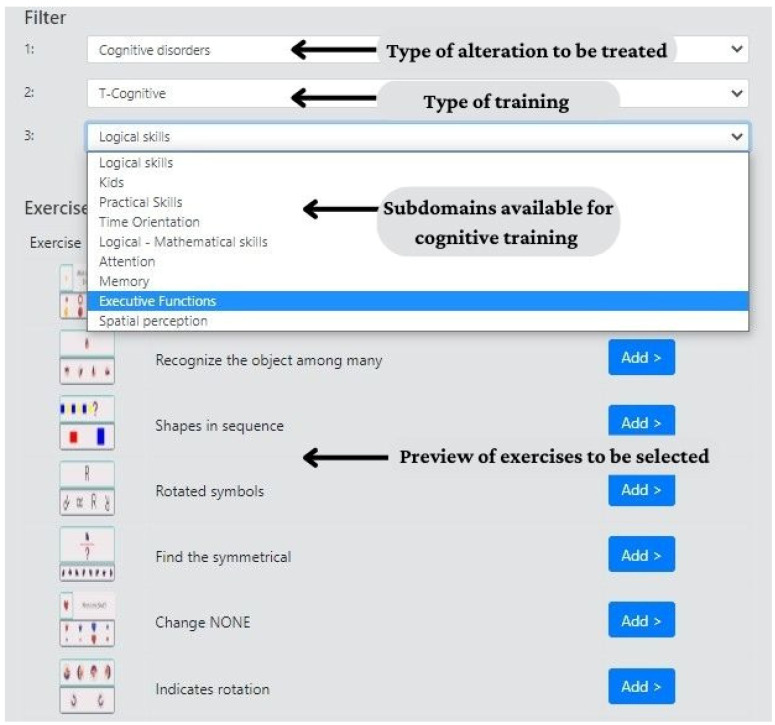
VRRS main screen showing the subdivision of the cognitive exercises into the various subdomains and categories.

During a VR-CT session, the patient is seated in front of the device and interacts with it actively, guided by the aid of the psychiatric-cognitive therapist. Notably, the VRRS contain forty-five different exercises and for each of these, the therapist can change the scenario of the virtual task, increasing its complexity and adding more distractors. The exercises already available are divided into specific subdomains (Figure 2), including orientation (personal/autobiographical, spatial/topographical and temporal), attention processes (selective attention, sustained attention and split attention), memory (verbal and visuo-spatial) and visual-executive tasks (verbal fluency, reasoning, categorization, problem solving and coping strategies). 

Each type of virtual exercise provided through the VRRS can be organized in 2 main criteria, differing in the way of interaction with the virtual reality tool. The 1st category includes 2D exercises where the patient interacts with objects and scenarios through the touch screen or through a particular magnetic tracking sensor coupled with a squeezable object, thus emulating mouse-like interaction capabilities. The 2nd category consists of 3D exercises, where the patients interact with 3D virtual scenarios and objects through magnetic wearable sensors generally placed over the hand (that permits a 3D position tracking of the end effector). 

## 3. Statistical Analysis

We performed a non-parametric statistical analysis according to the results from the Shapiro–Wilk test and the small size of the sample. Wilcoxon signed rank test was used to compare scores at baseline (T0) and at post-treatment (T1) for each group, while the Mann–Whitney test was performed to detect any statistical difference between the two groups (VR-CT and C-CT) at the onset of the study and on their post-intervention scores. Linear correlations were calculated with Spearman’s rank correlation coefficient (SCC). Each statistical operation was performed on R 4.1.3 for Windows (R Core Team (2022).) [29], a free software widely used for statistical computing, and interpreted at the two-tailed significance level of 0.05. In addition, we calculated the effect size (ES) using Glass’s delta, preferable for non-parametric and small sample sizes.

## 4. Results

At baseline (T0), we did not find any statistical difference in socio-demographic data and scores between the two groups (see Table 1). 

Comparing pre- and post- treatment scores, in the VR-CT group, we found significant differences in all outcome measures for global cognitive (MoCA: *p* < 0.005; 26.1 ± 3.14), executive (FAB: *p* < 0.005; 16.35 ± 1.6) and attention (TMT-A: *p* < 0.005; 60.2 ± 47.85; TMT-B *p* < 0.005; 172.7 ± 81.8; TMT-BA: *p* < 0.001; 111.9 ± 73.40) functioning, as well as in depressive symptoms HRS-D (*p* < 0.008; 7.2 ± 6.05). In addition, we detected statistically significant differences in anxiety (*p* < 0.008; 63.1 ± 17.39), general health (*p* < 0.02; 57.8 ± 18.56), vitality (*p* < 0.008; 55 ± 12.9), positive well-being (*p* < 0.005; 49.6 ± 20.52), self-control (*p* < 0.008; 58.8 ± 11.35), but also in coping strategies, including social support (*p* < 0.01; 24.5 ± 4.97), avoidance strategies (*p* < 0.005; 21.6 ± 2.79), positive attitude (*p* < 0.007; 29.7 ± 4.49) and problem solving (*p* < 0.01; 28.3 ± 3.3). In the C-CT group we found significant improvement at T1 in MoCA (*p* < 0.03; 24.3 ± 3.02), FAB (*p* < 0.02; 15.15 ± 2.15) and TMT-BA (*p* < 0.01; 226.3 ± 103.72), as well as in general health (*p* < 0.03; 54.3 ± 14.79), avoidance strategies (*p* < 0.01; 24.8 ± 2.97), positive attitude (*p* < 0.02; 24.6 ± 3.43) and problem solving (*p* < 0.008; 21.4 ± 7.01) (Table 4). 

Between-group post-treatment analysis revealed statistically significant differences only in the TMT-BA (*p* < 0.02, ES = 1.55) and COPE sub-items, including positive attitude (*p* < 0.02, ES = 1.13) and problem solving (*p* < 0.001, ES = 2.09) (Table 4).

Moreover, in the VR-CT group, we found two strong negative and linear correlations: between anxiety (PGWBI) and visuo-spatial/divided attention (TMT-B) rho = −0.66 and between depression (PGWBI) and visual processing skills (TMT-A) rho = −0.68. Indeed, the VR-CT group showed moderate and negative linear correlations between depressive symptoms (HRS-D) and frontal abilities (FAB) rho = −0.37 and between depressive symptoms (HRS-D) and visuo-spatial/divided attention (TMT-B) rho = −0.37. Lastly, in the experimental group, two moderate and positive correlations were found between self-control (PGWBI) and frontal abilities (FAB) rho = 0.50, as well as between problem solving (COPE) and visuo-spatial/divided attention (TMT-B) rho = 0.37.

## 5. Discussion

As far as we know, this is the first study that investigated the effects of non-immersive VR cognitive training on executive functions and coping strategies in patients with moderate to severe TBI. We found that all TBI patients receiving specific cognitive training improved their global cognitive and executive function, even though those treated with VR achieved better outcomes, including FAB.

The use of VR in the neurological field has been increasing in the last decades, demonstrating its efficacy in improving both motor and cognitive outcomes. A systematic review showed that the use of different VR tools may optimize cognitive abilities after TBI, with significant improvements also in executive function. Another more recent systematic review [30], involving five studies and two previous reviews, found that VR (either non immersive or immersive) may improve specific cognitive domains, including attention processes, working memory, visuo-spatial abilities and executive function. This further confirms how patients affected by TBI may benefit from cognitive rehabilitation using VR. 

Differently from previous works, we have used a VR training specifically designed to improve executive function, and more research is needed to confirm whether and to which extent a tailored cognitive training is fundamental also when VR exercises are applied.

On the contrary, in the study by Yip [31], no significant improvement was reported in both experimental and control groups, although there was weak evidence for the effects of VR cognitive training post TBI, especially in executive functioning domain.

The better outcomes in individuals undergoing VR may be explained by the fact that this may potentiate the “relearning”, also acting on executive processes. In fact, the use of augmented feedback and enjoyable training setting could influence neuroplastic processes, promoting cognitive recovery through motivation and increased collaboration and compliance which allows more intensive sessions [32,33]. Similar beneficial effects are being observed also by Navarro-Martos et.al, which investigated the effects of non-virtual games (a pie toy used in the intervention) in people with moderate Alzheimer Disease (AD). The authors confirm the positive role of a social and emotional climate to promote participants’ motivation and to stimulate executive functions [34]. 

In this vein, VR and its task-oriented approach can develop the knowledge of the results of movements (knowledge of results) and the knowledge of the quality of movements (knowledge of performance), gaining motor learning or relearning [35,36]. Thus, VR exercises could allow greater results than standard paper–pencil exercises, due to the global stimulation and dual cognitive and motor tasking [23]. It is noteworthy that VR tools differ on the degree of immersivity and interaction, and researchers have distinguished them in non-immersive and immersive virtual systems. In fact, non-immersive VR systems consist of standard 2D monitor exercises, while Computer Assisted Virtual Environment (CAVE) and head mounted display (i.e., the Oculus) use VR immersive interaction. In this way, VR offers a wide variety of rehabilitation settings based on type and severity of cognitive/motor dysfunctions and neurological disorder [30,37]. Given that the use of non-immersive VR led to positive outcomes in our sample, further studies should investigate the application of our TBI patient-tailored cognitive training using immersive VR.

In addition to the better improvement in cognitive functions, the VR-CT group showed good results also in coping strategies and problem solving, which is correlated with divided attention tasks. In line with our results, Rakers et al. showed strong correlations between executive functioning and coping styles in patients affected by moderate and severe TBI, reflecting that ED can complicate active coping strategies such as problem solving [5].

Examining the relationships between executive functioning, coping and depressive symptoms, it has been suggested that individuals who report greater difficulties with executive functioning after BI are inclined to use maladaptive passive coping styles [38]. TBI patients may benefit from a tailored rehabilitation approach which is directed to use more active and productive coping strategies [38]. In fact, improving coping strategies after TBI may help in potentiating both executive function and mood, given the correlations between these factors [39]. 

Notably, depressive symptoms improved in both experimental and control groups, but the outcomes reach a higher statistical significance in the experimental group, further demonstrating the role of VR in improving also behavioral symptoms. 

In line with our results, Sánchez-Nieto et al. have shown that VR-based therapy can produce behavioral and physiological changes also in AD patients [40], further confirming the increasing literature that supports this association in TBI subjects [41,42,43,44,45]. 

Moreover, we found a significant increase in positive attitude and problem solving in the VR group, and this may maintain motivation and confidence and potentially reduce depressive symptoms. Larger sample size studies are needed to confirm this important correlation. In this context, the neuromodulation approach, alone or combined to cognitive training (virtual and not), could be an innovative rehabilitative strategy to implement in the current clinical practice in relation to the patient’s needs, as suggested by Nousia et al. [46]. Owing to the suboptimum methodological quality of published studies, additional research is of potential value.

Our study has some limitations to acknowledge. The sample is relatively small to extend the results to the general TBI population; however, the study was conceived as pilot RCT and the number of subjects enrolled is in line with the study design. Another limitation was the allocation procedures that were not concealed, this should increase the risk of bias. Since the study has no follow up, we are not able to state if and to which extent the improvement lasts. It should be interesting to evaluate the long-term psychometric outcomes, in addition to the instrumental measures such as fMRI to investigate the neural plasticity. Moreover, although there is growing evidence on the positive effects of VR in the neurological field, the systematic use of VR in clinical practice is limited by two main factors: accessibility and the cost of virtual tools. 

## 6. Conclusions

To summarize, VR cognitive rehabilitation can be a promising tool to improve visuo-executive functions and coping strategies in patients affected by severe and chronic TBI. The VR can further potentiate neural plasticity through intensive, repetitive and motivating rehabilitation sessions, allowing recovery. The VRRS is equipped with a big screen and contains a wide range of virtual exercises divided in specific subdomains to perform a tailored cognitive training based on specific patients’ needs. 

This could be therefore be considered as a useful complementary treatment that potentially reduces depression symptoms providing enjoyment and active involvement of TBI patients, as confirmed by our results. This is why VR training should be implemented in current clinical practice, once confirmed by larger sample studies. 

## Figures and Tables

**Table 1:** Demographic and clinical description of the sample at the beginning of the study.

	All Participants	VR-CT	C-CT	*p*-Values
Age	44.6 ± 16.13	46.2 ± 14.9	43.1 ± 17.9	0.88
Gender				0.65
Male	11 (55.00)	5 (50.00)	6 (55.00)	
Female	9 (45.00)	5 (50.00)	4 (45.00)	
Education				0.70
Elementary	3 (15.00)	1 (10.00)	2 (20.00)	
Middle	3 (15.00)	1 (10.00)	2 (20.00)	
High school	9 (45.00)	6 (60.00)	3 (30.00)	
University	5 (25.00)	2 (20.00)	3 (30.00)	
Hemiparesis	11	6	5	
Tetraparesis	9	4	5	
Comorbidities				0.94
Diabetes	5	3	2	
Hypertension	8	4	4	
Heart diseases	5	2	3	
Chronic kidney diseases	2	1	1	
MoCA	22.4 ± 3.10	22.1 ± 2.84	22.7 ± 3.46	0.62
HRS-D	9.9 ± 6.34	8.5 ± 7.24	11.3 ± 5.29	0.65
TMT-A	100.05 ± 92.4	73.3 ± 57.4	124.8 ± 115.6	0.30
TMT-B	217.5 ± 98.2	207.8 ± 88.7	227.2 ± 110.8	0.84
TMT-BA	148.3 ± 112.4	132.5 ± 81.4	164.1 ± 139.5	0.88
FAB	14.1 ± 2.2	13.9 ± 2.3	14.3 ± 2.1	0.59
PGWBI				
Anxiety	67.25 ± 17.20	66.5 ± 17.43	68 ± 17.88	0.91
Depression	66.75 ± 19.62	65.1 ± 19.66	68.4 ± 20.5	0.67
General Health	42.4 ± 17.88	41.1 ± 18.27	43.7 ± 18.37	0.81
Vitality	44.5 ± 17.23	45 ± 16.15	44 ± 19.11	0.70
Positive well-being	37.75 ± 16.42	36.5 ± 15.1	39 ± 18.37	1
Self-control	55 ± 13.78	55.6 ± 11.59	54.4 ± 16.31	0.79
COPE				
Social support	21.9 ± 3.69	22.1 ± 3.95	21.7 ± 3.49	0.62
Avoidance strategies	23.2 ± 3.39	25.2 ± 2.85	21.2 ± 2.69	0.30
Positive attitude	25.9 ± 3.59	24.8 ± 3.58	27 ± 3.43	0.17
Problem solving	25.2 ± 2.62	24.9 ± 2.93	25.5 ± 2.41	0.56
Turning to religion	22.8 ± 3.80	23.2 ± 3.52	22.4 ± 0.70	0.67

Continuous variables are expressed as mean ± standard deviation, whereas categorical variables as frequencies and percentages. Legend: VR-CT (Virtual Reality Training), C-CT (Conventional Cognitive Training), MoCA (Montreal Cognitive Assessment), HRS-D (Hamilton Rating Scale—Depression), TMT (Trial Making Test), FAB (Frontal Assessment Battery) PGWBI (Psychological General Well-Being Index) COPE (Coping Orientation to the Problems Experiences-new Italian version).

**Table 2:** Cognitive Training focused on visuo-executive abilities: individual session, duration and type of treatment.

Rehabilitation Program	Intervention	Individual Session Duration	Type of Intervention	Exercise—Time	Cognitive Domains
Step 1 3 months Standard Neurorehabilitation (October–December 2021)	Conventional Cognitive Training (C-CT)	6 weekly sessions of 60 min (72 total treatments)	ROT (Reality Orientation Therapy) 20 min	10 min	Personal/Autobiographical Orientation
				5 min	Temporal orientation
				5 min	Spatial orientation
			Executive Training 40 min	10 min	Verbal Fluency (Phonemic and Semantic)/Categorization
				5 min	Working Memory
				10 min	Flexible Thinking/Attention Shifting
				15 min	Problem Solving /Reasoning/Coping Strategies
Step 2 3 months Advanced Rehabilitative Approach (January–March 2022)	Virtual Reality Rehabilitation System (Virtual Reality Rehabilitation System)– VR-CT Software and Tools dedicate for Cognitive Module VRRS—Evo	3 weekly sessions of 60 min (36 total treatments)	ROT (Reality Orientation Therapy) 20 min Executive Training 40 min (The same program as CCT)		

**Table 3:** Cognitive Training focused on visuo-executive processes, including both standard and virtual training.

Domain	Sub Domains	Standard Tasks	Virtual Exercises
Orientation	Personal Orientation	To see and choose the standard stimuli administered, including (i) photographs (about best friends, pets,…) which are emotionally meaningful for patients, (ii) audio-video materials, such as voice recordings of family, friends and colleagues; (iii) listen to music with emotional meaningful songs. (iv) to see favorite movie scenes or videos about personal life scenes (birth of children, significant and personal events of life …).	To see and choose the emotional virtual pictures—personal setting—biographic virtual photo (about home, wife, mother…). Using VRRS, integrated to the virtual system, listening to affective audio-video materials such as voice recordings of family…; music tracks—emotionally meaningful songs; main list of favorite movie scenes; videos of personal life scenes (birth of children, significant personal event of life…)
	Topographical Orientation	To promote spatial orientation through memories and recalling of places, cities or streets, using ad hoc paper and pencil material. Administration of visuo-spatial tasks, spatial awareness exercises; realizing some traditional puzzles, 2D element’s position (center, right—left); drawing activity; and recognizing shapes and spatial relations.	To promote spatial orientation in a virtual space, to stimulate topographical sense and perception, using reasoning activities through the recognition of places, cities and different locations. Administration of virtual orientation tasks, spatial awareness activities with the execution of virtual Puzzles or virtual element’s position (center, right—left), virtual drawing activity or paint to explore interactive maps and shapes.
	Temporal Orientation	To increase temporal orientation ability through the repetition and recalling of specific information, such as personal data, personal events, with face-to-face activities to manage information relating to days, time, month. In this activity, the TBI patient must tell the time, day, month, year and current season, selecting which month they’re currently in at the time of doing the exercise.	To increase temporal orientation ability through the repetition and recalling of specific information, such as personal data, personal events, managing information related to days, time, month, using VVRS and virtual environment. The information is repeatedly transmitted through VR visuo-verbal, written or auditory modality.
Attention Processes	Selective Attention	The therapist administered some selective and double tasks such as selecting/associating the color to the dimension, or specific shape and simultaneously with the elimination of the different standard stimuli-target for an increasing time of execution	The therapist administered some selective and double tasks such as selecting/associating the color to the dimension or specific shape and simultaneously with the elimination of the different virtual stimuli-target for an increasing time of execution.
	Sustained attention		
	Split Attention		
Memory Abilities	Verbal	To stimulate verbal memory using mnemonic techniques and strategic skills through paper and pencil materials and a series of traditional tasks such as trying to recall the words of a song or a written text, poetry after reading, or songs and books, according to a classic approach.	To stimulate verbal memory using mnemonic techniques and strategic skills through a pc—based task and virtual exercises such as trying to recall the words of a song or a written text, poetry after reading, using VRRS system and pc-verbal based auditory tasks.
	Non-Verbal/Visuo-spatial	To stimulate visual memory skills, TBI patients must tap on the traditional cards, matching pairs of pictured objects or peoples’ photographs. To support non-verbal auditory memory skills, patients tap on the cards matching commonly heard sounds.	To stimulate visual memory skills, TBI patients must tap on virtual cards, matching pairs of pictured objects or virtual photographs of people. To support non-verbal auditory memory skills, patients tap on virtual cards, matching pairs of common sounds heard in a virtual environment using VRRS system.
Executive Functions	Verbal Fluency	To promote the recovery of executive functioning, the psychiatric therapist asks to the TBI patient to realize specific activities to stimulate categorization skills, semantic and phonemic categorization; activities planning and logical association; tasks of analogical reasoning, using paper and pencil tools.	To promote the recovery of executive functioning, the psychiatric therapist asks to the TBI patient to realize specific activities to stimulate categorization skills, semantic and phonemic categorization; activities planning and logical association; tasks of analogical reasoning using a pc-based approach and virtual 2D and 3D activities.
	Reasoning		
	Categorization		
	Coping Strategies	The therapist invites the patient to build sequential logical sequences using standard cards with colorful images representing animals, money, and objects. Then the patient has to order them according to variable criteria. The therapist asks the patient to find a solution to a problem of daily life through a conventional methods.	The therapist invites the patient to build sequential logical sequences using virtual game cards with colorful images representing animals, money and objects and to order them according to variable criteria. The therapist asks the patient to find a solution to a problem of daily life using a virtual tool.
	Problem Solving		

**Table 4:** Statistical comparison of clinical score variations from baseline to follow-up between experimental group (VR-CT) and control group (C-CT). Significant *p*-values are in bold.

Outcome Measure	Intra-Group Analysis		Between Group Analysis (Post-Treatment Comparison)	Effect Size (ES) (Glass Delta)
	VR-CT	C-CT		
MoCA	0.005	0.03	0.25	0.57
FAB	0.005	0.02	0.24	0.84
HRS-D	0.008	0.02	0.47	0.31
TMT-A	0.005	0.16	0.09	0.73
TMT-B	0.005	0.9	0.30	0.55
TMT-BA	0.001	0.01	**0.02**	1.55
PGWBI				
Anxiety	0.008	0.28	0.30	0.38
Depressed mood	0.07	0.57	0.32	0.41
General Health	0.02	0.03	0.54	0.18
Vitality	0.008	0.20	0.07	0.77
Positive well-being	0.005	0.28	0.05	1.79
Self-control	0.008	0.15	0.42	0.37
COPE				
Social support	0.01	0.06	**0.70**	0.84
Avoidance strategies	0.005	0.01	0.05	1.14
Positive attitude	0.007	0.02	0.02	1.13
Problem solving	0.01	0.008	0.001	2.09
Turning to religion	1	0.88	0.67	0.21

## Data Availability

Data will be available on-demand to the corresponding author.
